# Collapsing singletons may boost signal for associating rare variants in sequencing study

**DOI:** 10.1186/1753-6561-8-S1-S50

**Published:** 2014-06-17

**Authors:** Wei Wang, Zhi Wei

**Affiliations:** 1 Department of Computer Science, New Jersey Institute of Technology, University Heights Newark, New Jersey 07102, USA

## Abstract

Advances in next-generation sequencing technology have made it possible to comprehensively interrogate the entire spectrum of genomic variations including rare variants. They may help capture the remaining genetic heritability which has not been fully explained by previous genome-wide association studies. Here we performed a gene-based genome-wide scan to identify hypertension susceptibility loci in analysis of a whole genome sequencing cohort of 103 unrelated individuals. We found that collapsing singletons may boost signals for associating rare variants and identified *SETX *statistically significant by a genome-wide gene-based threshold (*p *value <5.0 × 10^−6^). The function of *SETX *in hypertension may be worthy of further investigation.

## Background

Assuming the "common disease-common variant" hypothesis, genome-wide association studies (GWAS) have successfully identified hundreds of common variants that contribute to human traits and diseases [1]. These common variants, however, account for only a small fraction of disease or trait heritability. On the other hand, rare variants, having minor allele frequencies (MAFs) between 0.1% and 1%, may be functionally relevant and causal for a larger proportion of inheritable variability [2,3].

Recent advances in next-generation sequencing (NGS) technology have made it technically and economically feasible to capture the full spectrum of genomic variation. NGS provides a powerful tool for systematic exploration of common and rare variants in the entire genome, even in large population-scale studies [4]. However, pinpointing causal variants remains a major challenge, particularly for associating rare variants with complex traits [5]. There is a substantial need for computational methods that allow for efficient association analysis of rare variants. Several powerful approaches tailored for rare-variant association studies have been proposed [6-9]. These tests offer us a powerful tool to investigate rare variants in the entire genome.

## Methods

### Data set

The Genetic Analysis Workshop 18 (GAW18) provided a whole genome sequencing data set for a hypertension cohort of 483 individuals. These samples were sequenced by Complete Genomics with approximately 60× coverage, and odd-numbered autosomes data were made available for analysis. After quality control, 464 individuals and 24 million single-nucleotide polymorphisms (SNPs) remained. Of those SNPs, more than 51% had MAFs <1%, which was the focus of our analysis in this article. The longitudinal hypertension phenotypes were provided for up to 4 time points. Because our analysis was focused on binary traits, we treated individuals diagnosed with hypertension in any of the 4 times as cases. We extracted 103 genetically unrelated individuals with both phenotype data and sequencing data. We found 39 unaffected controls and 64 cases affected by hypertension.

### Preprocessing

The variants were stored in VCF files. We preprocessed them as follows. To end up with rare variants, we filtered out SNPs that were present in dbSNP132 or MAFs >1%. We also filtered out SNPs with a genotype missing rate >5%. The remaining missing genotypes were resampled from nonmissing individuals.

$$X_{i}=\left\{\begin{array}{cc} 1, & if\;any\;x_{i,j}=1\\ 0, & otherwise \end{array}\right)$$

Next, we grouped variants into sets based on RefSeq gene annotations [10], requiring SNPs lie between the RefSeq transcript start site and transcript end site. SNPs outside gene boundaries were not analyzed. In total, 10,148 genes from odd-numbered chromosomes were used. Finally, we collapsed singletons within each gene. A singleton was a variant being observed only once among all the samples. The rationale of collapsing singletons was that the distribution of singletons as 1 variable may reflect the association between genotype and phenotype. Hence, we created 1 supervariant for each gene by combining all the singletons within it using the following rules: for each sample, (a) the genotype was set to be 1, if there was at least 1 variant observed; (b) otherwise, the genotype was set to be 0.

### Rare-variant association tests

We employed 3 recently published rare association tests, qMSAT [7], C-alpha [8], and CMC [9]. The qMSAT is a quality-weighted multivariate score association test that can utilize genotype quality information. However, genotype quality score information was not available in the GAW18 raw VCF files. Without utilizing quality information, the qMSAT test was equivalent to the linear sequence kernel association test (SKAT) [6], Sum of Squared U statistic test (SSU) [11], and C-alpha. The C-alpha test compared the assumed binomial distribution of rare variants in cases versus controls via a homogeneity test. CMC, a combined multivariate and collapsing method, collapsed variants in subgroups according to allele frequencies and combines these subgroups using Hotelling's *T^2 ^*test. For these 3 tests, we used permutation to evaluate association significance. Because permutation was computationally expensive, we utilized a 2-step strategy in searching and testing candidate loci. Specifically, we first used 1000 permutations, from which we can identify candidates with estimated *p *value <0.001. Then for these candidates we conducted 10^6 ^permutations so as to know if any loci were significant at a genome-wide gene-based threshold (0.05/10,000 = 5.0 × 10^−6^) using a Bonferroni assumption.

## Results

After the preprocessing step, we obtained approximately 2.2 million rare variants, which were assigned to 10,148 genes for testing. We then performed the 3 tests, qMSAT, C-alpha, and CMC, using R (http://www.r-project.org). The qMSAT, C-alpha, and CMC identified 10, 6, and 7 genes with an estimated *p *value <0.001, respectively (Table 1). Only SETX revealed significance for all of the 3 methods. Using 10^6 ^permutations, qMSAT, C-alpha, and CMC yielded more precise *p *values of 2.0 × 10^−6^, 1.0 × 10^−6^, and 6.0 × 10^−6^, respectively, for SETX (Table 2). The CMC *p *value was slightly higher than the genome-wide gene-based threshold, which was possibly a result of its lower power compared to qMSAT and C-alpha [7].

**Table 1:** Genes with *p *<0.001 from at least 1 method using 10^3 ^permutations


**Chr**	**Gene**	**# Variants (singletons)**	**qMSAT**	**C-alpha**	**CMC**

chr1	NUP210L	674(221)	Y		
chr1	USP1	51(19)	Y	Y	
chr7	CUL1	348(114)	Y		
chr9	RAB14	88(32)	Y		
chr9	**SETX**	380(135)	Y	Y	Y
chr11	FLJ39051	44(11)	Y		
chr11	GDPD5	338(104)	Y		
chr19	GRIN3B	24(8)	Y		
chr19	LOC100505495	249(78)	Y	Y	
chr19	PSG5	111(26)	Y		Y
chr5	CXXC5	96(35)		Y	
chr15	RCCD1	32(13)		Y	
chr17	WSCD1	183(70)		Y	
chr17	MLLT6	92(33)			Y
chr1	ATF6	576(173)			Y
chr7	ZNF775	69(21)			Y
chr19	LOC100128252	45(13)			Y
chr5	LOC728342	495(146)			Y

**Table 2:** Functional annotation and test of the rare variants in SETX


		***p *Value | OR | 95% CI**			*p *Value*		

**Regions**	**# Variants (singletons)**	**Fisher's exact test on supervariant^†^**			**qMSAT**	**C-alpha**	**CMC**

**SETX (CDS + UTR + INTRON)**	380(135)	3.7 × 10^−6^	8.8	[3.12, 27.43]	2.0 × 10^−6^	1.0 × 10^−6^	6.0 × 10^−6^
**CDS**	14(8)	1.000	1.0	[0.18, 6.94]	1.000	0.544	0.837
**UTR**	6(4)	0.632	0.6	[0.04, 8.60]	1.000	0.662	0.990
**INTRON**	360(123)	8.8 × 10^−7^	9.5	[3.43, 28.70]	<1.0 × 10^−6^	<1.0 × 10^−6^	<1.0 × 10^−6^

*p Values were calculated using 10^6 ^permutations.

^†^Supervariant was defined by collapsing all the singletons.

SETX locates in chr9:135,136,827-135,230,372 and is a relatively large gene among all the human genes. The length of SETX (93,545 base pairs [bp]) is far greater than the median number (17,970 bp) of all the genes (*p *value <2.2 × 10^−16^, one-sided Wilcoxon signed rank test). Although it contains 26 exons, the total length of coding regions is only 8,034, suggesting that SETX includes large intronic regions. To pinpoint causal regions, we divided the 380 variants of SETX into 3 groups based on its functional annotations. Specifically, we applied ANNOVAR [12] to annotate the variants of SETX and grouped them into coding sequence regions (CDSs), untranslated regions (UTRs), and intronic regions (INTRONs) (see Table 2). We observed that a majority of rare variants were, indeed, from the intronic region. We tested these 3 regions using the same tests with 10^6 ^permutations. We found that the UTR group and the CDS group were far from being significant, suggesting that they may be irrelevant. Another possible reason may be that there are very few variants in these categories. Because the INTRON group became more significant than the whole gene-based tests after excluding the variants from these 2 groups, we may conclude that causal variants locate in the intronic region of SETX.

We then sought to elucidate why and where the signal came from. To this end, several in-depth analyses for SETX were performed. First, the Fisher's Exact test was conducted on the super feature we created by collapsing singletons. We found that, by collapsing all the 135 singletons on SETX, it achieved a very significant *p *value (3.7 × 10^−6^), together with OR = 8.8 and 95% CI = [3.12, 27.43] (see Table 2). This explained why SETX could be detectable under such a small sample size. We obtained more significance when testing the super feature with only singletons within the intronic regions (*p *value = 8.8 × 10^−7^, OR = 9.5, and 95% CI = [3.43, 28.70]), which was consistent with the results from 3 rare variant association tests. Second, we checked each rare variant and singleton individually by performing the same test. It turned out that none of them were significant, when the minimum *p *value was merely 0.14. This demonstrated that the significance of SETX was very unlikely a result of technical artifact, such as systematic sequencing error or imputation bias, because the new feature was a combination of hundreds of singletons. It also highlighted that collapsing singletons may increase power when studying association of rare variants using a relatively small sample size. Third, we took a closer examination of allele frequencies of the 380 rare variants located in SETX. Of the rare variants, 92 could be found in the 1000 Genomes Project (2012 February release, http://www.1000genomes.org/). We found their allele frequencies in general population were extremely low (mean frequency = 0.0004 for 92 rare variants), indicating that these variants were so rare that they may collectively have a composite effect of OR = 8.8 while missed in previous studies.

Finally, to further remove possible confounding effect of population stratification, we performed a principal component analysis on 100,000 randomly selected common variants with no missing value and MAF >0.1. Logistic regression test was then conducted on the created super feature for SETX, together with the first 10 principal components as covariates. We found that the super feature remained significant, with a *p *value = 6.7 × 10^−5^, while the 10 principal components were not.

The protein encoded by SETX contains a DNA-RNA helicase domain at its C-terminal, suggesting its involvement in both DNA and RNA processing. Mutations in SETX have been reported to be associated with ataxia-ocular apraxia-2 (AOA2) [13] and an autosomal dominant form of juvenile amyotrophic lateral sclerosis (ALS4) [10,14]. However, the function of SETX and its role in hypertension remains unclear and may be worthy of further investigation.

## Discussion

We performed 3 rare-variant association tests for the analysis of a whole genome sequencing data set to identify susceptibility genes in hypertension. We grouped and collapsed rare variants in a gene-based manner for 2 reasons: (a) the deleteriousness of variants could come from protein-coding sequence changes or noncoding intronic regions that contain regulatory elements. (b) Based on the previous simulation study [7], the power of the analysis could be as low as 0.2 (sample size <200). By collapsing singletons, one may benefit from increasing power. This idea was essentially similar as those burden tests for rare copy number variation in GWAS and de novo mutations in sequencing study. Indeed, we found that the signal was mainly from the intronic regions of SETX in a collective manner of those singletons.

The analysis can be extended and improved in several ways. First, it was shown in qMSAT [7] that incorporation of genotype call qualities directly in association tests can increase power. If raw reads are available, we may call variants and obtain genotype quality information at the same time, using variant calling toolkits [15,16] to further increase our analysis power. Second, we only analyzed rare variants. The association test could be also performed by combining both rare and common variants. Third, we only included 103 unrelated individuals. We may consider adding more samples to increase power. Finally, we focused on only genic regions using conventional gene annotation, which make up little more than 1% of the genome. The recent annotation made by the ENCODE consortium has included more than 70,000 "promoter" regions and nearly 400,000 "enhancer" regions that regulate expression of distant genes, which account for roughly 80% of the genome [17]. We may utilize this new knowledge in future analysis.

## Competing interests

The authors declare that they have no competing interests.

## Authors' contributions

WW conducted statistical analyses; WW and ZW drafted the manuscript. All authors read and approved the final manuscript.
